# Response of Non-Structural Carbohydrates in *Picea crassifolia* to Elevation Gradients in the Qilian Mountains

**DOI:** 10.3390/plants15192984

**Published:** 2026-09-30

**Authors:** Min Ma, Ni Wang, Ning Liu, Xuee Ma, Yurui Tang

**Affiliations:** 1 College of Forestry, Gansu Agricultural University, Lanzhou 730070, China18810228030@163.com (N.L.); 2 Academy of Water Resource Conservation Forests in Qilian Mountains of Gansu Province, Zhangye 734000, China; maxuee111@126.com; 3 Key Laboratory of Arid Climatic Change and Disaster Reduction of Gansu Province, Key Laboratory of Arid Climatic Change and Disaster Reduction of China Meteorological Administration, Institute of Arid Meteorology, China Meteoro-Logical Administration, Lanzhou 730020, China; 18419060862@163.com

**Keywords:** non-structural carbohydrates, elevation gradient, source-sink relationship, alpine coniferous forest, *Picea crassifolia*, Qilian Mountains

## Abstract

Non-structural carbohydrates (NSC) are important components of plant photosynthetic carbon products and play key roles in carbon allocation and stress adaptation. We hypothesized that elevational gradients and organ functional differences jointly shape NSC concentrations in *Picea crassifolia*. We measured NSC, soluble sugar, and starch in leaves, trunks, and fine roots at three elevations (2500, 2900, 3300 m) in the Qilian Mountains. NSC and its components decreased with elevation in all organs, while the soluble sugar-to-starch ratio increased. NSC concentrations ranked leaves > fine roots > trunks, and the relative distribution of starch shifted from trunks at low elevations to leaves at high elevations. Climate and soil jointly explained 79.70% of NSC variation, with the elevational-climate gradient being the dominant driver. These findings enhance understanding of carbon allocation strategies in alpine coniferous forests.

## 1. Introduction

Non-structural carbohydrates (NSC) are substances temporarily stored following photosynthesis, and their reserves can reflect the level of available energy for plant growth, survival, and resistance to environmental stress [1,2]. NSC is mainly composed of soluble sugars (SS) and starch (ST), which are interconvertible and co-regulate the plant’s carbon supply–demand relationship, thereby maintaining carbon homeostasis [3,4]. Specifically, soluble sugars serve as rapidly cycled carbon sources that directly participate in respiration and growth processes, while also playing key roles in osmotic regulation, low-temperature adaptation, and stress resistance. Starch, as the primary storage form, can be hydrolyzed and reconverted into available carbon sources during resource-limited periods, providing energy for maintaining basal metabolism and resuming growth [5]. NSC dynamics not only reflect the balance between carbon acquisition and consumption in plants but also indicate their ability to adjust carbon allocation strategies under environmental stress.

Different plant organs possess distinct structural components and functional properties, and their NSC concentrations reflect the plant’s internal carbon allocation and functional trade-off mechanisms. Leaves are important organs for plants to perform photosynthesis and obtain resources from the aboveground environment [6]. As a perennial woody organ, the trunk not only provides mechanical support but also serves as a critical transport channel connecting carbon sources and sinks, playing an important role in carbon storage and maintaining plant adaptation to environmental stress [7]. Fine roots are responsible for absorbing water and mineral nutrients, and they are important organs for plants to obtain underground resources and maintain growth [8]. Due to functional differentiation among organs in carbon acquisition, transport, storage, and consumption, NSC concentrations and component ratios often exhibit significant organ specificity. Analyzing NSC concentrations at the organ level can help reveal how plants coordinate carbon storage, growth requirements, and environmental adaptation, providing deeper insight into plant resource utilization strategies and ecological adaptation mechanisms [9,10].

Mountain ecosystems exhibit significant environmental heterogeneity and serve as natural experimental platforms for studying the ecological adaptation mechanisms of plants [11]. As an important topographic factor, elevation influences plant growth and development, biomass accumulation, and the distribution patterns of NSC by altering regional water and heat conditions and the availability of soil resources. Currently, two hypotheses explain the variation in plant NSC along elevational gradients: the carbon limitation hypothesis and the growth limitation hypothesis. The carbon limitation hypothesis posits that low temperatures at high elevations reduce photosynthetic carbon assimilation, resulting in insufficient carbon input and thereby restricting NSC synthesis and accumulation [12,13]. By contrast, the growth limitation hypothesis suggests that low temperatures mainly inhibit plant growth and carbon consumption, so that carbon assimilation exceeds consumption, causing excess photosynthetic products to be stored as NSC [14,15]. Relevant studies have shown that these two hypotheses are not mutually exclusive but rather coexist across different plant life forms, varying levels of environmental stress, and seasonal dynamics, jointly determining the balance among carbon acquisition, utilization, and storage [16]. Apart from temperature, moisture and soil nutrients are also key factors driving NSC dynamics. Along elevational gradients, plants may encounter different types of resource limitations. For instance, in low-elevation areas they may experience water stress, while in high-elevation areas they are more limited by low temperatures and shorter growing seasons. These environmental pressures jointly influence plant carbon acquisition and allocation strategies [17]. When nutrient supplies such as nitrogen and phosphorus are limited, plants may adjust carbon allocation patterns, prioritizing resources to structural growth and defense, thereby affecting NSC accumulation [4]. Furthermore, plants can regulate the conversion between soluble sugars and starch to balance rapid carbon utilization against long-term storage, thus forming distinct carbon storage strategies [1,18]. Along such elevational gradients, multiple environmental factors covary and confound one another. It should be noted that field elevational gradients represent complex, multifactorial systems involving temperature, precipitation, soil properties, phenology, and genetic variation. Purely observational field surveys cannot isolate the causal effect of any single environmental factor on plant NSC. Accordingly, the present study characterizes observed patterns and explores correlative relationships rather than testing causal mechanisms. Nevertheless, characterizing these observed patterns is a critical first step toward understanding plant adaptation strategies and provides an important foundation for future experimental research. Given this context, studying NSC concentrations in *P. crassifolia* across elevations is essential for understanding carbon investment trade-offs between plant growth and defense.

The Qilian Mountains are located in the transitional zone between the Qinghai–Tibet Plateau cold region and the northwestern desert region of China. Given their unique geographical location and ecological position, they constitute an important ecological security barrier in northwestern China [19]. Driven by complex terrain, hydrothermal conditions, and habitat heterogeneity, vegetation types, community structures, and ecological functions in the Qilian Mountains exhibit significant horizontal and vertical gradients [20]. *P. crassifolia*, as the dominant coniferous species in the forest ecosystem of the Qilian Mountains, plays a crucial role in regional forest formation, carbon cycle regulation, water retention, and biodiversity maintenance [21]. However, the cold and arid habitat conditions restrict the growth and natural regeneration of *P. crassifolia*, rendering the forest ecosystem in this region particularly sensitive to environmental changes and human disturbances. Therefore, elucidating the NSC pattern of *P. crassifolia* under different environmental conditions is essential for understanding its environmental adaptation mechanisms and maintaining the stability of regional forest ecosystems. At present, research on *P. crassifolia* has mainly focused on stand dynamics and regeneration [22], soil nutrient characteristics and ecological stoichiometry [23,24], hydrological functions and climate response mechanisms [25,26], ecosystem services [27,28], and the impacts of pest and disease disturbances [29,30]. These studies have provided an important foundation for understanding the ecological functions and environmental response processes of *P. crassifolia*. Nevertheless, knowledge of NSC patterns in *P. crassifolia* remains limited in three key aspects. First, most previous studies have focused on a single organ and have rarely compared NSC responses across multiple organs (leaf, trunk, fine root). Second, the relative contributions of soluble sugar and starch to elevational changes, and organ-specific shifts in their proportions, are poorly understood. Third, the relative importance of climate versus soil factors in driving NSC variation along elevational gradients has not been quantified for this species. Therefore, investigating organ-level NSC concentrations in *P. crassifolia* along an elevational gradient can help reveal the carbon balance regulation mechanisms and resource adaptation strategies of this species in high-altitude environments.

This study focused on *P. crassifolia* at different elevations in the Qilian Mountains, and measured the concentrations of NSC and its components in leaves, trunks, and fine roots. Given the functional differences among organs in carbon acquisition, transport, and storage, as well as the impact of environmental changes associated with the elevational gradient on plant carbon balance, we hypothesized that organ functional differentiation and the environmental gradient jointly shape the patterns of NSC and its components in *P. crassifolia*, thereby enabling the species to develop carbon storage strategies adapted to different habitat conditions. Based on this premise, this study aims to address two scientific questions: (1) Do different organs of *P. crassifolia* exhibit distinct NSC concentration patterns? (2) How do environmental factors along the elevational gradient correlate with NSC variation in *P. crassifolia*? This study enhances our understanding of NSC patterns and environmental adaptation mechanisms of dominant tree species in alpine forests, and provides a scientific basis for the protection and sustainable management of forest ecosystems in the Qilian Mountains.

## 2. Results

### 2.1. The Variation Characteristics of Soil Physical and Chemical Properties of P. crassifolia at Different Altitudes

**Figure 1:**
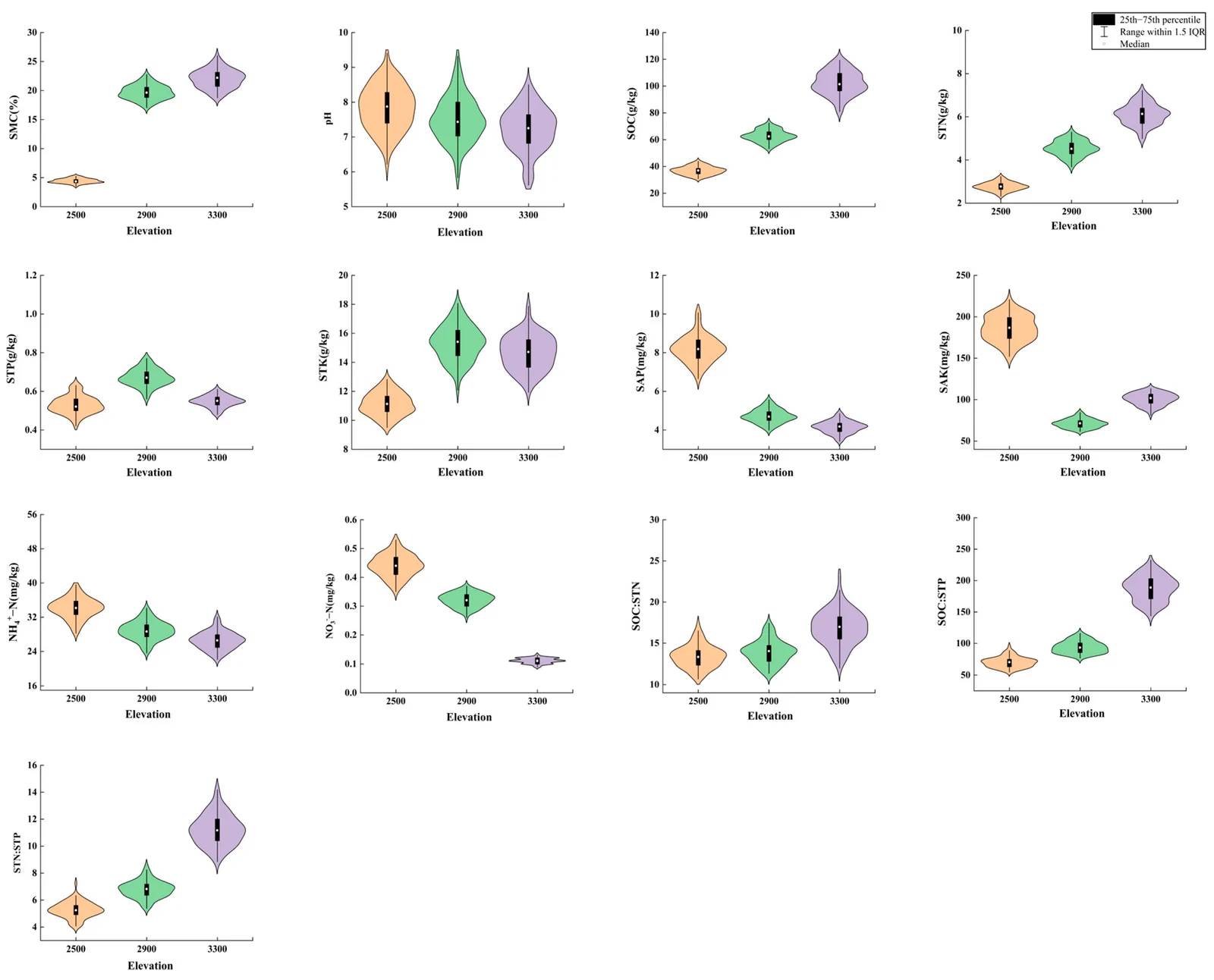
Characteristics of soil physicochemical properties at different elevations. SMC: soil moisture content; SOC: soil organic carbon; STN: soil total nitrogen; STP: soil total phosphorus; STK: soil total potassium; SAP: soil available phosphorus; SAK: soil available potassium; NH_4_^+^-N: soil ammonium nitrogen; NO_3_^−^-N: soil nitrate nitrogen; SOC:STN: the ratio of SOC to STN; SOC:STP: the ratio of SOC to STP; STN:STP: the ratio of STN to STP.

As shown in Figure 1, soil moisture content (SMC), soil organic carbon (SOC), soil total nitrogen (STN), and the SOC:STN, SOC:STP, and STN:STP ratios increased with elevation by factors of 5.0, 2.78, 2.21, 1.26, 2.68, and 2.13, respectively. In contrast, soil pH decreased from 7.83 at 2500 m to 7.21 at 3300 m, while available phosphorus (SAP), ammonium nitrogen (NH_4_^+^-N), and nitrate nitrogen (NO_3_^−^-N) decreased by 49.39%, 22.26%, and 75.00%, respectively. Soil total phosphorus (STP) and total potassium (STK) showed hump-shaped patterns with elevation, ultimately increasing by 3.77% and 32.07%, respectively. Soil available potassium (SAK) showed the opposite pattern, ultimately decreasing by 45.97%.

### 2.2. Variation in NSC and Its Component Concentrations Among Organs of P. crassifolia at Different Elevations

Linear mixed-effects models revealed that elevation, organ type, and their interaction had highly significant effects on NSC, soluble sugar, starch, and the soluble sugar-to-starch ratio (all *p* < 0.001; Table 1). Marginal R^2^ values ranged from 0.969 to 0.989, indicating that fixed effects explained nearly all of the observed variance.

**Figure 2:**
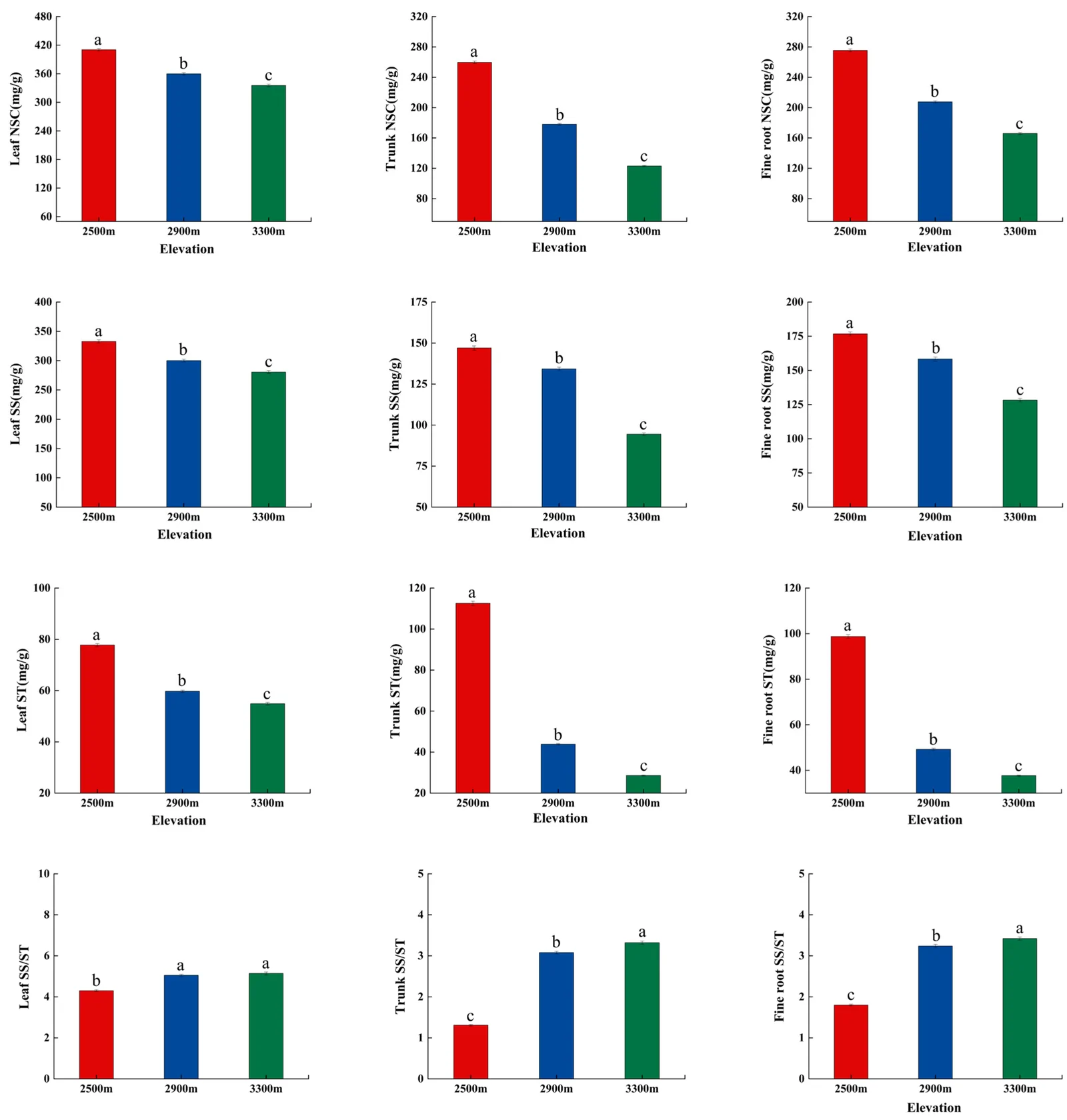
Changes in NSC and its component concentrations in various organs of *P. crassifolia* at different elevations. NSC: non-structural carbohydrates; SS: soluble sugar; ST: starch; SS/ST: the ratio of soluble sugar to starch. Different uppercase letters indicate significant differences among elevations within the same organ, while different lowercase letters indicate significant differences among organs at the same elevation.

The NSC and its component concentrations in different organs of *P. crassifolia* differed significantly among elevations (*p* < 0.05; Figure 2). With increasing elevation, NSC concentrations in leaves, trunks, and fine roots decreased by 18.31%, 39.76%, and 52.59%, respectively. Both soluble sugar and starch concentrations in the three organs were significantly higher at low elevations than at high elevations, with starch showing a more pronounced decline than soluble sugar across all organs. In contrast, the soluble sugar-to-starch ratio across all organs was significantly lower at low elevations than at high elevations, and increased by 0.20-, 0.90-, and 1.53-fold in leaves, trunks, and fine roots, respectively (Figure 2).

**Figure 3:**
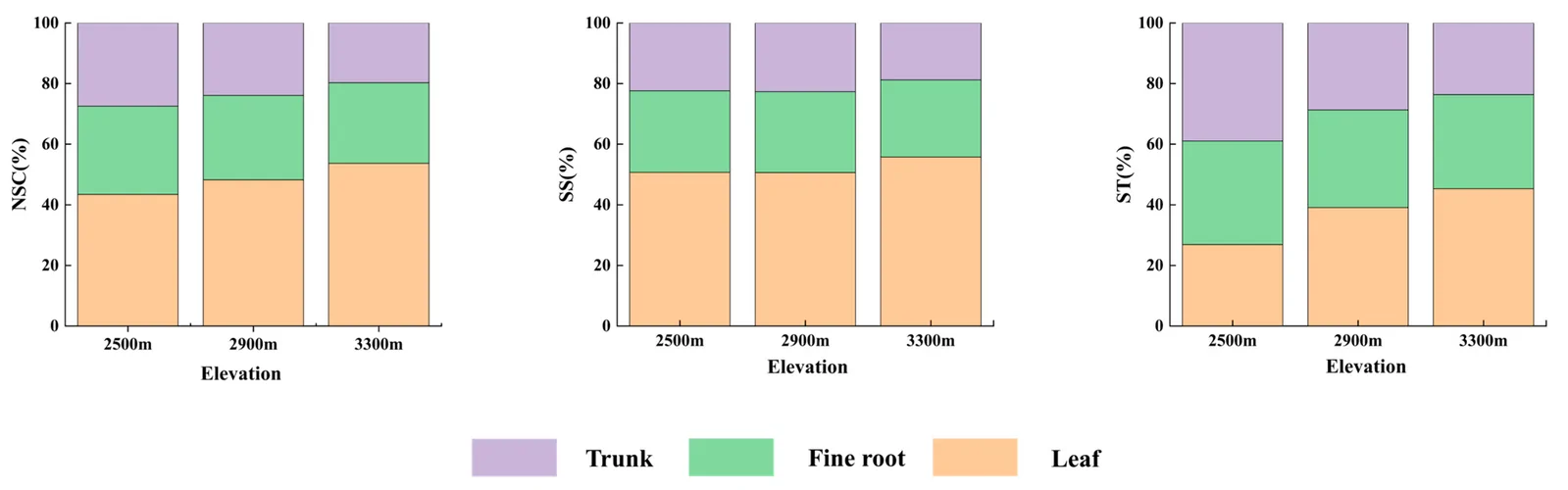
Distribution patterns of NSC and its component concentrations in various organs of *P. crassifolia* at different elevations. NSC: non-structural carbohydrates; SS: soluble sugar; ST: starch.

Across the elevational gradient, NSC concentrations were consistently highest in leaves (accounting for >40% of the total across organs), followed by fine roots and trunks (Figure 3). The relative proportion of NSC in leaves increased with elevation. Notably, the organ distribution of starch shifted with elevation. At low elevations, starch concentrations were highest in trunks, whereas at high elevations they were highest in leaves (Figure 3).

### 2.3. Effects of Environmental Factors on NSC and Its Component Concentrations in P. crassifolia Along an Elevational Gradient

Changes in plant NSC concentrations are influenced by environmental factors and can reflect the adaptive strategies adopted by plants in response to environmental changes. To investigate the effects of environmental factors on NSC concentrations in *P. crassifolia*, this study used RDA and PCA.

**Figure 4:**
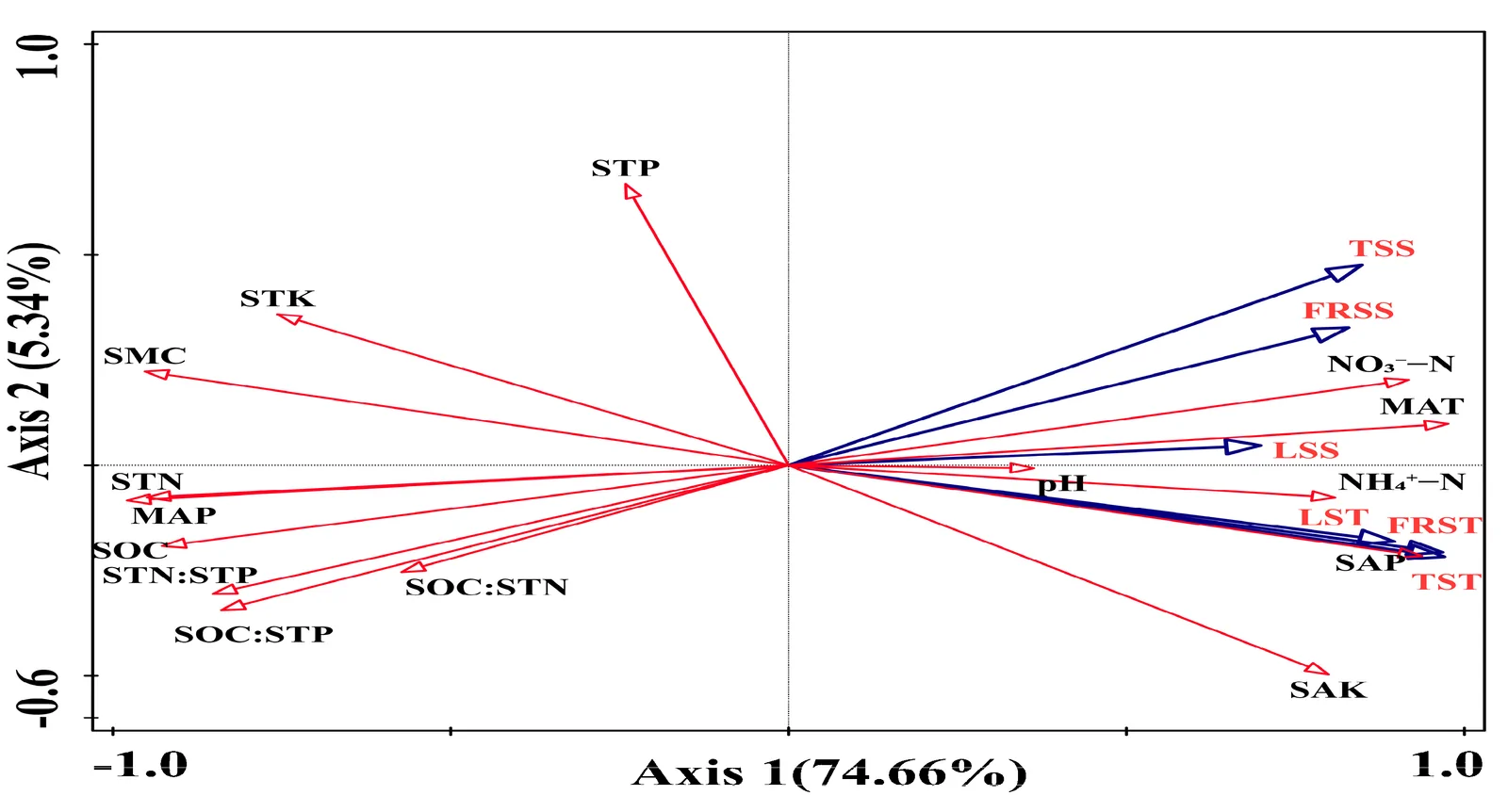
Redundancy analysis (RDA) of NSC composition in various organs of *P. crassifolia* and environmental factors. Red arrows represent environmental factors, and blue arrows represent soluble sugar and starch contents in leaves, trunks, and fine roots. SMC: soil moisture content; SOC: soil organic carbon; STN: soil total nitrogen; STP: soil total phosphorus; STK: soil total potassium; SAP: soil available phosphorus; SAK: soil available potassium; NH_4_^+^-N: soil ammonium nitrogen; NO_3_^−^-N: soil nitrate nitrogen; LSS: leaf soluble sugar; LST: leaf starch; TSS: Trunk soluble sugar; TST: Trunk starch; FRSS: Fine root soluble sugar; FRST: Fine root starch; SOC:STN: the ratio of SOC to STN; SOC:STP: the ratio of SOC:STP; STN:STP: the ratio of STN to STP; MAT: mean annual temperature; MAP: mean annual precipitation.

RDA results (Figure 4) showed that the first and second ordination axes jointly accounted for 80.00% of the total variation in NSC concentrations, with MAT and MAP exerting the most significant effects on all NSC components in *P. crassifolia* (*p* < 0.05). Variation partitioning indicated that climate and soil factors together explained 79.70% of the total variation in NSC (adjusted R^2^ = 0.797, *F* = 64.287, *p* < 0.001). The unique contribution of climate was 0.94% (*p* < 0.001), whereas that of soil was only 0.19% (*p* = 0.200), with the shared fraction reaching 78.55%. This suggests high collinearity between climate and soil.

**Figure 5:**
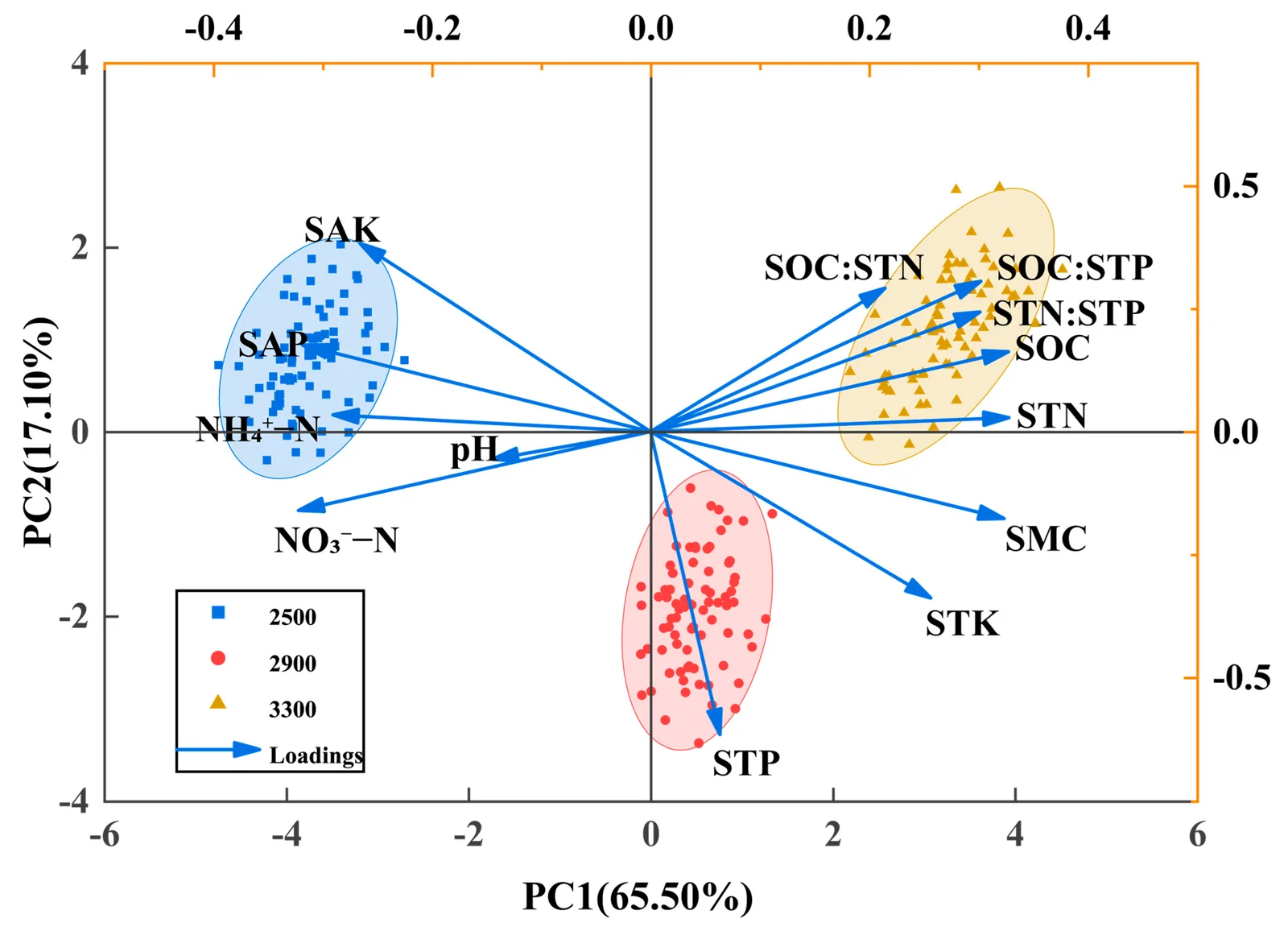
Principal component analysis (PCA) of soil factors for *P. crassifolia* at different elevations. SMC: soil moisture content; SOC: soil organic carbon; STN: soil total nitrogen; STP: soil total phosphorus; STK: soil total potassium; SAP: soil available phosphorus; SAK: soil available potassium; NH_4_^+^-N: soil ammonium nitrogen; NO_3_^−^-N: soil nitrate nitrogen; SOC:STN: the ratio of SOC to STN; SOC:STP: the ratio of SOC:STP; STN:STP: the ratio of STN to STP.

Given the multicollinearity among the 13 soil factors, PCA-based dimensionality reduction was performed. PCA results (Figure 5) showed that PC1 was highly positively correlated with SOC, STN, and SMC, but negatively correlated with SAP and nitrate nitrogen (NO_3_^−^-N), characterizing a decoupling gradient between high organic matter pools and low nutrient availability, and was thus designated as the Soil Fertility Index (SFI). PC2 was positively correlated with STP and STK, but negatively correlated with SAK and the SOC:STP ratio, representing the transformation dynamics between soil phosphorus-potassium total pools and their availability, and was designated as the Phosphorus-Potassium Transformation Index (PKI). The first two principal components cumulatively explained 82.56% of the total soil variation.

## 3. Discussion

### 3.1. Variation in NSC and Its Component Concentrations Among Different Organs of P. crassifolia Across Elevations

Elevation-mediated variation in abiotic environments is a key driver of plant growth, community composition, and ecosystem processes [31]. The long-standing debate regarding elevational patterns of tree NSC centers on two competing hypotheses: the carbon limitation hypothesis, which posits that low temperatures restrict photosynthetic carbon gain leading to insufficient carbon supply, and the sink limitation (or growth limitation) hypothesis, which suggests that low temperatures suppress growth more strongly than photosynthesis, resulting in carbon accumulation [15]. Hoch and Körner found that trees at the alpine treeline maintain similar or even higher NSC concentrations compared to conspecifics at lower elevations, supporting the sink limitation hypothesis [14,32]. Our results show that NSC concentrations in *P. crassifolia* declined consistently with elevation across all organs, with starch declining more sharply than soluble sugars (Figure 2), partially supporting the carbon limitation hypothesis during the mid-growing season. This discrepancy with previous global patterns likely arises because our elevational gradient does not reach the climatic alpine treeline, and the semi-arid climate of the Qilian Mountains imposes additional drought and nutrient constraints on carbon gain that are absent from the more humid alpine systems where most classic treeline studies were conducted. Within this regional context, Shi et al. [33] and Qin et al. [34] reported lower NSC concentrations at higher elevations in *P. crassifolia* in the Qilian Mountains, with the latter showing a shift from growth limitation at lower elevations to carbon limitation at higher elevations. In drier mountain systems, low temperatures may coincide with periodic water limitation and low nutrient availability, both of which can independently restrict photosynthetic carbon gain, potentially pushing the system toward carbon limitation even below the climatic treeline.

The low temperatures accompanying increasing elevation may directly inhibit Rubisco activity, potentially leading to reduced photosynthetic rate and carbon assimilation efficiency. If photosynthetic carbon input is insufficient to offset metabolic costs, NSC reserves would be expected to decline, consistent with the carbon limitation hypothesis [35]. As the primary form of long-term carbon storage, starch may be preferentially hydrolyzed under carbon limitation to sustain respiratory metabolism. This preferential depletion of starch over soluble sugars under carbon-limiting conditions has been widely documented, suggesting it is a generalized physiological response rather than a species-specific trait. In line with this mechanism, the decline in starch was more pronounced than that in soluble sugars across all organs [36]. Additionally, although high-elevation soils have accumulated higher organic carbon and total nitrogen, low temperatures may suppress microbial mineralization rates, leading to a decoupling between total soil nutrient pools and plant-available nutrient supply [37]. This decoupling is notable because elevated soil C and N stocks at high elevation do not necessarily translate into greater nutritional availability for trees, a point often overlooked in NSC elevation studies that rely solely on total soil nutrient measures. Insufficient available nitrogen could further constrain Rubisco synthesis and carbon assimilation, though this mechanism remains speculative given that we did not directly measure soil nitrogen mineralization rates.

The soluble sugar to starch ratio increased with elevation across all organs. We acknowledge that sampling was conducted in mid-August during the growing season, prior to winter dormancy. Therefore, interpretations regarding cold acclimation should be considered speculative. Several alternative mechanisms, all operating during the growing season, may explain this pattern. Among these, reduced sink demand likely plays the central role, as low temperatures suppress cambial activity and cell division more strongly than they constrain photosynthetic carbon fixation, thereby reducing starch deposition in growing tissues and leaving a higher proportion of newly fixed carbon as soluble sugars [38]. Ongoing starch mobilization may also support higher respiratory demand at cooler high-elevation temperatures, while soluble sugars may be preferentially retained as metabolic substrates and osmolytes during the growing season itself [5]. The relative contributions of these mechanisms remain unresolved and would require combined measurements of cambial growth, respiration, and sugar–starch turnover across seasons. The elevated ratio at high elevations does not necessarily indicate winter cold adaptation, but rather reflects a growing-season carbon balance between source supply and sink demand.

In *P. crassifolia*, NSC and soluble sugars were consistently highest in leaves, followed by fine roots and trunks (Figure 3). This pattern aligns with the functional heterogeneity of different organs, as leaves are the primary source organs for photosynthetic carbon assimilation, where newly fixed carbon is initially formed as soluble sugars such as sucrose [3]. Soluble sugars function not only as direct substrates for phloem transport and respiratory metabolism, but also participate in osmotic adjustment. Fine roots are important carbon sink organs that require a continuous supply of soluble sugars to sustain respiration, growth, and nutrient uptake, which explains why their NSC concentration is second only to that of leaves [9]. The trunk, which primarily functions in mechanical support and water conduction, maintains relatively lower NSC concentrations. Prior research on *P. crassifolia* has similarly shown that soluble sugar, starch, and NSC levels follow the order leaves > fine roots > twigs, attributed to leaf photosynthetic function and carbohydrate accumulation [21].

Notably, starch concentrations in trunks were higher than in leaves at low elevations but lower at high elevations (Figure 3). We discuss this pattern with caution, as it is based on concentration data (mg/g dry mass) rather than total pool size, and cannot be interpreted as a true biomass allocation shift. This relative shift in starch concentration distribution may be associated with several factors. At low elevations, higher temperatures and a longer growing season support vigorous cambial activity, potentially driving greater photosynthate transport to trunks for accumulation as storage starch. With increasing elevation, low temperatures may suppress cambial carbon demand more strongly than they limit leaf carbon assimilation, leading to relatively higher starch concentrations remaining in leaves. However, we acknowledge that this interpretation is speculative and requires further investigation with multi-seasonal sampling and biomass measurements to validate.

### 3.2. Environmental Drivers of NSC and Its Component Concentrations in P. crassifolia Along Elevational Gradients

RDA and variance partitioning indicate that NSC patterns in *P. crassifolia* are primarily structured by the elevational climate gradient (MAT and MAP), with climate and soil jointly accounting for the majority of the observed variation. Air temperature and precipitation directly drive photosynthetic carbon gain and growing season length, while simultaneously shaping soil temperature, moisture, and nutrient availability. The large shared fraction therefore reflects the integrated nature of the elevational environmental gradient rather than the independent influence of any single factor. Unlike manipulative experiments, observational gradient studies inherently cannot separate these co-varying drivers.

Soil PCA further revealed a decoupling between total soil nutrient pools and nutrient availability. PC1 was positively correlated with SOC, STN, and SMC, but negatively correlated with SAP and NO_3_^−^-N, indicating that higher soil organic carbon and total nitrogen do not necessarily translate into higher available nutrient supply (Figure 5). At high elevations, lower temperatures may reduce organic matter decomposition and microbial mineralization rates, potentially facilitating the accumulation of soil organic carbon and total nitrogen while plant-available nutrients remain low [37,39]. This finding carries an important methodological implication for elevational NSC research, as studies that rely solely on total soil C and N as explanatory variables may misrepresent the nutritional environment experienced by trees. Plant-available nutrient measures should be incorporated alongside total pools to better capture soil nutrient effects on tree carbon balance. This decoupling between high total nutrient content and low availability could contribute to the large shared explained variance of climate and soil factors, though we acknowledge that microbial processes were not directly measured in this study. Soil samples from three depth layers (0–10, 10–20, 20–40 cm) were combined into a single 0–40 cm composite for this analysis, chosen because fine roots of *P. crassifolia* are primarily distributed within this depth range; future studies should examine vertical soil stratification in greater detail.

The spatial distribution of NSC in *P. crassifolia* appears to be co-regulated by the elevational-climate gradient and local soil conditions. The elevational gradient is associated with the general decrease in NSC components in all organs, while local soil conditions may further modulate starch accumulation in sink organs such as trunks and fine roots. Soil moisture and available nutrients may influence leaf nitrogen nutrition and photosynthetic capacity, thereby altering photosynthate input, while also regulating carbon allocation by modulating cambial growth and fine root development. Soil conditions may exert a stronger effect on starch than on soluble sugars, which can be attributed to the distinct functional roles of these two carbohydrate classes. Starch serves as a relatively stable storage compound whose accumulation depends on the carbon surplus between photosynthetic supply and growth costs [3]. Soluble sugars, in contrast, possess multiple functions as metabolic substrates, transport compounds, and osmoregulatory substances, and their concentrations are coordinately regulated by sugar–starch conversion and inter-organ transport [5,9]. This physiological buffering may render soluble sugar concentrations less responsive to local soil variation. Starch reserves, which dominate long-term carbon storage in woody tissues, may respond more strongly to changing soil moisture and nutrients than soluble sugars, suggesting that soil-mediated effects will shape future elevational NSC patterns.

## 4. Materials and Methods

### 4.1. Study Site

**Figure 6:**
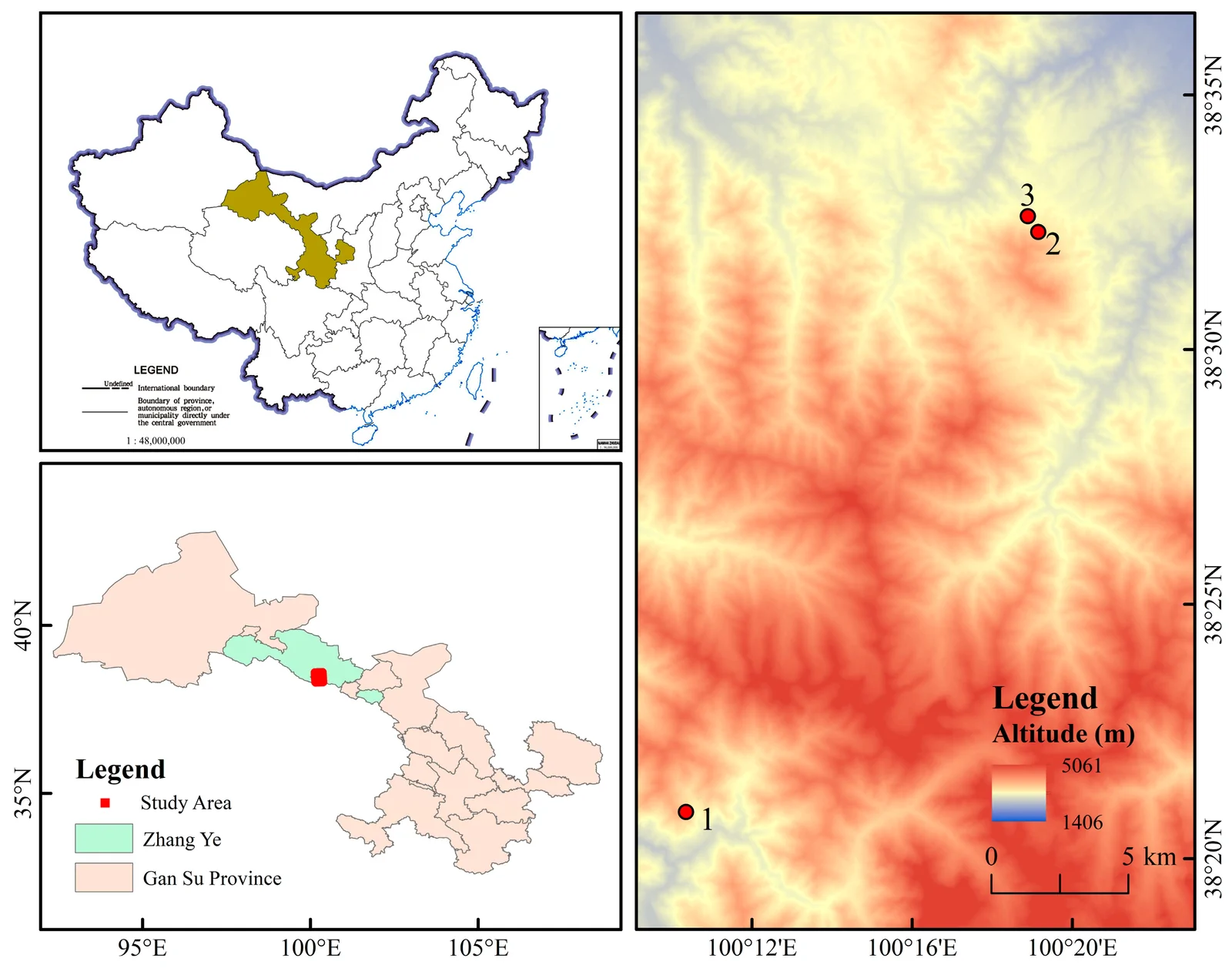
Location of study area and field sampling plots.

The sampling sites were located in the Pailugou watershed of the Xishui forest region, in the central Qilian Mountains of Gansu Province (37°28′–39°04′ N, 97°20′–102°13′ E) (Figure 6; plots 1–3 correspond to elevations of 2500 m, 2900 m, and 3300 m, respectively). This region is characterized by an alpine semi-arid montane forest-steppe climate with significant vertical climatic zonation [27]. The mean annual temperature is 1.7 °C, mean annual evaporation is 923.1 mm, mean annual sunshine duration is 1595.6 h, mean daily total radiation is 110.28 kW·m^−2^, and the mean annual relative humidity is 59%. The main soil types include mountain chestnut soil, mountain gray-cinnamon soil, subalpine shrub meadow soil, and alpine cold desert soil. *P. crassifolia*, the dominant species of Qilian Mountains forests, is mainly distributed on shaded and semi-shaded slopes at elevations of 2400–3300 m [40]. Dominant shrub species include *Potentilla fruticosa*, *Caragana jubata*, and *Salix gilashanica*, while dominant herbaceous species mainly comprise *Polygonum viviparum*, *Carex atrata*, and *Stipa* spp. [34].

### 4.2. Plot Establishment and Sample Collection

In August 2024, nine sample plots were established across three elevations (2500, 2900, and 3300 m) based on the distribution characteristics of *P. crassifolia*, the constructive species of the region, with three randomly arranged plots at each elevation (Table 2). Each plot measured 20 m × 20 m. To minimize the effects of variations in light and needle surface temperature on carbohydrate content, all sampling was conducted at noon [41]. It should be noted that this single-timepoint sampling was performed during the mid-growing season, representing NSC concentrations at the seasonal growth peak rather than year-round patterns. In each plot, nine upright, healthy, undamaged, and non-isolated *P. crassifolia* trees of uniform size (age, tree height, and DBH) were randomly selected, and leaf, trunk, and fine root (diameter < 2 mm) samples were collected. Leaf samples were collected from unshaded, mature one- and two-year-old branches on the upslope side and pooled. Trunk samples were obtained by extracting wood cores at breast height (1.3 m) using an increment borer (CO400, Haglöf Sweden AB, Långsele, Sweden). After removing the bark, the entire core was retained. Sapwood and heartwood were not physically separated. Fine root samples (<2 mm in diameter) were collected by excavation at soil depths of 5–30 cm. After being assigned unique identification numbers, the samples were immediately placed in a cooler for field storage and transported back to the base that same day, where they were heated in a microwave oven (P70D20TL-D4, Galanz, Foshan, China) for 90 s [42]. Finally, all samples were dried at 65 °C in a forced-air drying oven (MOV-212F, Panasonic, Osaka, Japan) until they reached a constant weight for subsequent NSC analysis [43].

### 4.3. Determination of NSC Content

The dried leaf, trunk, and fine root samples were separately ground in a high-speed universal grinder (FW100, Tianjin Taisite Instruments Co., Ltd., Tianjin, China) and passed through a 2 mm mesh sieve for analysis. The contents of soluble sugars and starch were determined using anthrone colorimetry [44], with the absorbance measured using a UV-V is spectrophotometer (UV-2600, Techcomp, Shanghai, China). NSC content was calculated as the sum of soluble sugar and starch contents.

### 4.4. Measurement of Soil Physicochemical Properties

In each plot, soil samples were collected from the center and four corners at depths of 0–10 cm, 10–20 cm, and 20–40 cm. After removing stones, gravel, and plant residues, samples from the same depth were thoroughly mixed, and the three depth layers were combined in equal masses into a single 0–40 cm composite representing the overall soil conditions in each plot. For all subsequent RDA and PCA analyses, the 0–40 cm integrated soil composite was used. Soil moisture content was determined by the oven-drying method [45]. Soil pH was measured using the potentiometric method [19] with a pH meter (PHS-3C, INESA Scientific Instrument, Shanghai, China) Soil organic carbon was determined using the potassium dichromate oxidation-heating method [46]. Soil total nitrogen was determined using the Kjeldahl method [35]. Soil total phosphorus and available phosphorus were analyzed by the molybdenum-antimony colorimetric method [47]. Soil total potassium and available potassium were determined using the flame photometric method [48] with a flame photometer (6400A, Shanghai Precision Instruments, Shanghai, China). Soil ammonium nitrogen and nitrate nitrogen were extracted with potassium chloride and measured [49].

### 4.5. Data Analysis

The mean annual temperature and precipitation were calculated as follows [31,50]:

MAT = 20.957 − 5.49 × 10^−3^*H* − 0.166*X* + 0.89 × 10^−2^*Y   R*^2^ = 0.98 (1)

MAP = 1680.6235 + 0.119*H* − 75.264*X* + 12.405*Y   R*^2^ = 0.92 (2)

where MAT is the mean annual temperature, MAP is the mean annual precipitation, *H* is the elevation, *X* is the latitude, *Y* is the longitude, and *R*^2^ is the regression coefficient.

Linear mixed-effects models (LMMs) were used to test the effects of elevation, organ type, and their interaction on NSC and its component concentrations in *P. crassifolia*. Elevation and organ type were included as fixed factors, while plot and tree identity were included as random effects to account for spatial dependence and the nested sampling design. Kenward-Roger’s method was used to estimate denominator degrees of freedom, and Type III Wald chi-square tests were performed to assess the significance of fixed effects. Tukey-adjusted pairwise comparisons were conducted using estimated marginal means (emmeans) to identify significant differences among elevations within each organ and among organs at each elevation. Redundancy analysis (RDA) was employed to explore the relationships between NSC components and environmental factors, with forward selection used to identify significant variables and variation partitioning applied to quantify the relative contributions of climatic and soil factors. To address multicollinearity among the 13 soil factors, principal component analysis (PCA) was performed for dimensionality reduction, and the extracted principal components were retained as comprehensive soil indices. All statistical analyses were conducted in R 4.4.1 (R Foundation for Statistical Computing, Vienna, Austria) using the lme4, lmerTest, and emmeans packages and Canoco 5.0 (Biometris, Wageningen University and Research, Wageningen, the Netherlands). Figures were generated using Origin 2024b (OriginLab Corp., Northampton, MA, USA).

## 5. Conclusions

As important carbon reserve substances in plants, NSC concentrations not only determine carbon supply for growth and metabolism, but also serve as key physiological indicators of plant adaptation to environmental stress. This study investigated changes in NSC and its components among different organs of *P. crassifolia* across an elevational gradient during the mid-growing season. Increasing elevation reduced NSC, soluble sugar, and starch concentrations in all organs, while the soluble sugar-to-starch ratio increased. NSC and soluble sugar concentrations were consistently highest in leaves, followed by fine roots and trunks. Notably, the relative distribution of starch shifted from trunks at low elevations to leaves at high elevations. Variation partitioning revealed that climate and soil factors collectively explained 79.70% of NSC variation, with the elevational-climate gradient being the dominant driver. Several limitations should be acknowledged. Single-timepoint sampling in mid-August reflects mid-growing-season conditions rather than annual NSC dynamics, and trunk wood cores were not separated into sapwood and heartwood. Future studies should incorporate multi-seasonal sampling, in situ microclimate monitoring, and sapwood-specific NSC measurements, as well as controlled experiments to disentangle temperature vs. soil effects.

## Figures and Tables

**Table 1:** Linear mixed-effects model of NSC concentrations in *P. crassifolia* across elevation and organ type.

	Soluble Sugar		Starch		NSC		Soluble Sugar/Starch	
	F	*p*	F	*p*	F	*p*	F	*p*
Elevation (E)	661.77	<0.001	6381.39	<0.001	2227.08	<0.001	876.19	<0.001
Organ (O)	9283.45	<0.001	13.31	<0.001	8183.28	<0.001	2585.85	<0.001
E × O	11.84	<0.001	655.35	<0.001	67.77	<0.001	58.54	<0.001

**Table 2:** Basic characteristics of the sampling sites.

Sample Site	Elevation (m)	Longitude	Latitude	Slope (°)	MAT (°C)	MAP (mm)	Canopy Density (%)	Mean Tree Height (m)	Mean Diameter at Breast Height (cm)	Mean Crown Width (m)
1	2500	100°17′24″ E	38°34′3″ N	34	1.72	330.38	76.70 ± 2.63 a	18.41 ± 0.45 a	24.80 ± 0.32 a	6.47 ± 0.11 a
2	2900	100°17′59″ E	38°32′27″ N	12	−0.47	380.14	66.70 ± 0.63 b	11.29 ± 0.30 b	18.77 ± 0.49 c	4.47 ± 0.18 b
3	3300	100°18′16″ E	38°32′9″ N	36	−2.67	427.13	63.30 ± 1.54 b	10.24 ± 0.19 c	21.65 ± 0.59 b	6.20 ± 0.11 a

MAT: mean annual temperature; MAP: mean annual precipitation. Different lowercase letters denote significant differences among sample sites at *p* < 0.05 (Tukey’s HSD test).

## Data Availability

The data used in the present work have been listed in the Supplementary Materials.

## Supplementary Materials

The following supporting information can be downloaded at https://www.mdpi.com/article/10.3390/plants15192984/s1: Table S1: Characteristics of soil physicochemical properties at different elevations (mean ± SE); Table S2: Non-structural carbohydrates (NSC) and its component concentrations in various organs of *P*. *crassifolia* at different elevations (mean ± SE).
