# Supplementary material for: Response of Non-Structural Carbohydrates in Picea crassifolia to Elevation Gradients in the Qilian Mountains
Source: Plants (Basel). 2026 Sep 30;15(19):2984. doi: 10.3390/plants15192984 (PMC13666853; doi:10.3390/plants15192984)
Supplement: Supplementary file 1 [file plants-15-02984-s001.zip › plants-4537367-supplementary.pdf]

Table S1 Characteristics of soil physicochemical properties at different elevations (mean ± SE).

Elevation (m)	SMC	pH	SOC (g/kg)	STN (g/kg)	STP (g/kg)	STK (g/kg)	SAP (mg/kg)	SAK (mg/kg)	NH_4^+-N (mg/kg)	NO_3^--N	SOC:STN	SOC:STP	STN:STP
2500	4.40±0.044c	7.83±0.071a	36.76±0.341c	2.76±0.022c	0.53±0.005c	11.1±0.088c	8.18± 0.077a	185.83± 1.755a	34.09± 0.268a	0.44± 0.004a	13.37± 0.153c	70.00± 0.926c	5.26± 0.065c
2900	19.69±0.140b	7.50±0.073b	62.62±0.493b	4.53±0.041b	0.67±0.005a	15.32± 0.135a	4.71± 0.039b	71.08±0.603c	28.84± 0.243b	0.32± 0.003b	13.90± 0.158b	93.87± 1.072b	6.78± 0.071b
3300	21.99±0.179a	7.21±0.069c	102.27± 1.000a	6.11±0.055a	0.55± 0.003b	14.66± 0.133b	4.14± 0.035c	100.41± 0.808b	26.5± 0.229c	0.11± 0.001c	16.86± 0.231a	187.35± 2.248a	11.19± 0.127a

Different lowercase letters in the same column indicate significant differences among plots (*P* < 0.05). SMC: soil moisture content; SOC: soil organic carbon; STN: soil total nitrogen; STP: soil total phosphorus; STK: soil total potassium; SAP: soil available phosphorus; SAK: soil available potassium; NH_4^+-N: soil ammonium nitrogen; NO_3^--N: soil nitrate nitrogen.

Table S2 Nonstructural carbohydrates NSC and its components in various organs of *P. crassifolia* at different altitudes (mean ± *SE*).

Elevation (m)	LSS (mg/g)	FRSS (mg/g)	TSS (mg/g)	LST (mg/g)	FRST (mg/g)	TST (mg/g)	LNSC (mg/g)	FRNSC (mg/g)	TNSC (mg/g)	LSS/ST	FRSS/ST	TSS/ST
2500	332.74±2.61a	176.70±1.55a	147.00±1.33a	77.76±0.58a	98.73±0.88a	112.57±1.06a	410.50±2.63a	275.43±1.74a	259.57±1.85a	4.30±0.05b	1.80±0.02c	1.31±0.02c
2900	300.07±2.43b	158.35±1.43b	134.24±1.11b	59.72±0.44b	49.24±0.43b	43.84±0.32b	359.79±2.48b	207.59±1.41b	178.07±1.16b	5.05±0.05a	3.24±0.04b	3.08±0.03b
3300	280.43±2.61c	128.26±1.25c	94.46±0.81c	54.88±0.52c	37.66±0.29c	28.61±0.26c	335.32±2.70c	165.92±1.34c	123.07±0.85c	5.14±0.07a	3.42±0.04a	3.32±0.04a

Different lowercase letters in the same column indicate significant differences among plots (*P* < 0.05). LSS: leaf soluble sugar; FRSS: Fine root soluble sugar; TSS: Trunk soluble sugar; LST: leaf starch; FRST: Fine root starch; TST: Trunk starch.
